# Uterine infusion of conceptus fragments changes the protein profile from cyclic mares

**DOI:** 10.1590/1984-3143-AR2020-0552

**Published:** 2020-11-25

**Authors:** Cesar Augusto Camacho, Gabriel de Oliveira Santos, Jorge Emilio Caballeros, Nicolas Cazales, Camilo José Ramirez, Pedro Marcus Pereira Vidigal, Humberto Josué de Oliveira Ramos, Edvaldo Barros, Rodrigo Costa Mattos

**Affiliations:** 1 Laboratório de Reprodução Animal, Faculdade de Veterinária, Universidade Federal do Rio Grande do Sul, Porto Alegre, RS, Brasil; 2 Facultad de Veterinária, Universidad de la República – UDELAR, Montevideo, Uruguay; 3 Núcleo de Análise de Biomoléculas, Universidade Federal de Viçosa, Viçosa, MG, Brasil

**Keywords:** maternal recognition, two-dimensional electrophoresis, mass spectrometry, embryo-maternal communication

## Abstract

This experiment aimed to compare at day seven after ovulation, the protein profile of uterine fluid in cyclic mares with mares infused two days before with Day 13 conceptus fragments. Experimental animals were ten healthy cyclic mares, examined daily to detect ovulation (Day 0) as soon as estrus was confirmed. On day seven, after ovulation, uterine fluid was collected, constituting the Cyclic group (n = 10). The same mares were examined in the second cycle until ovulation was detected. On day five, after ovulation, fragments from a previously collected concepti were infused into each mare's uterus. Two days after infusion, uterine fluid was collected, constituting the Fragment group (n = 10). Two-dimensional electrophoresis technique processed uterine fluid samples. A total of 373 spots were detected. MALDI-TOF/TOF and NanoUHPLC-QTOF mass spectrometry identified twenty spots with differences in abundance between the Cyclic and Fragment group. Thirteen proteins were identified, with different abundance between groups. Identified proteins may be related to embryo-maternal communication, which involves adhesion, nutrition, endothelial cell proliferation, transport, and immunological tolerance. In conclusion, conceptus fragments signalized changes in the protein profile of uterine fluid seven days after ovulation in comparison to the observed at Day 7 in the same cyclic mares.

## Introduction

The establishment and maintenance of pregnancy are critically dependent on embryo-maternal communication during the preimplantation period (Klein and Troedsson, 2011a). The crosstalk between the conceptus and reproductive tract results in the maintenance of progesterone production by the ovaries and preparing the uterus for embryo implantation (Waclawik et al., 2017). Equine early pregnancy is poorly understood (Swegen et al., 2017) because its reproductive physiology shows remarkable differences with other domestic species (Abd-Elnaeim et al., 2006; Klein, 2016). Thereupon, the conceptus-derived pregnancy recognition signal has not been identified (Klein and Troedsson, 2011b).

Many approaches have been used to improve understanding of the stage of the estrous cycle, uterine receptivity, embryo-maternal communication, and embryo metabolism. Thus, histomorphometric (Keenan et al., 1987; Caballeros et al., 2019; Camozzato et al., 2019) and molecular analysis (Merkl et al., 2010; Klein and Troedsson, 2011b; Swegen et al., 2017; Smits et al., 2018; Bastos et al., 2019) have been performed using equine endometrial tissues, uterine fluid, and embryos or conceptus fragments.

Infusion of conceptus fragments into the mare uterus influenced endometrial and vascular changes, with an increase of glandular secretion, suggesting that proteins present in the conceptus can alter uterine fluid (Camacho et al., 2018). Those endometrial changes raise the number of immune cells and differentiate cellular patterns similar to those observed in pregnant mares (Keenan et al., 1987; Camozzato et al., 2019).

It is hypothesized that the infusion of conceptus fragments promotes changes in the content of uterine fluid protein. This experiment aimed to compare at day seven after ovulation, the protein profile of uterine fluid in cyclic mares with mares infused two days before with 13^th^ day conceptus fragments.

## Materials and methods

### Animals

The study was performed during the southern hemisphere-breeding season. Experimental animals were ten healthy cyclic Quarter Horse type mares (mean age, 6.8, and range between 4 to 10 years old) weighing 450-550 Kg and kept in natural pastures with free access to mineral supplementation and water. Throughout the experiment, mares remained healthy and had an average body condition score of 3.5 (scale 1 to 5) (Malschitzky et al., 2001). This study was carried out, with an Animal Ethical Use Committee approved protocol at Universidade Federal do Rio Grande do Sul, Porto Alegre, Rio Grande do Sul, Brazil (protocol number 34572).

Mares were examined for reproductive soundness by evaluation of perineal conformation, transrectal palpation, ultrasound of genital tract (Sonoscape S8V, China), and endometrial biopsy. Only cyclic and clinically normal mares, with endometrium classified as category I or IIA (Kenney and Doig, 1986), without evidence of endometritis, were selected. All mares had previously foaled.

### Experimental design

Mare’s reproductive tracts were routinely examined by transrectal palpation and ultrasonography until estrus was detected. Estrus confirmed (ovarian follicle > 35 mm in diameter and marked uterine edema), mares were examined daily to detect ovulation, considered Day 0. In this first cycle, uterine fluid samples were collected on day seven after ovulation in all mares (n = 10). These mares constituted the Cyclic group.

In the second cycle, The same mares were examined during the second estrous cycle until ovulation was detected (Day 0). On Day 5, after ovulation, fragments from concepti recovered previously from pregnant mares were infused into the uterus of each mare. Uterine fluid samples were collected on Day 7 in all mares (n = 10). These mares constituted the Fragment group.

### Conceptus fragments

Conceptus fragments were obtained from previous embryo collections, performed on day 13 after ovulation, snap-frozen in liquid Nitrogen with 2.5 mL of Ringer solution. Before their infusion in the Fragment group mares, concepti were thawed at room temperature, where they collapsed due to their osmolality and size. Rapid cooling to temperatures as low as -70 °C prevents protein denaturation, maintaining its structure sufficiently well, and enabling normal enzymatic catalysis to proceed (Adams et al., 2015). Thawed concepti were divided equally into two parts. Each part was transferred to a Petri dish, diluted with Ringer solution to 2 mL, homogenized, and infused to each mare (n = 10). Infusion of fragments was carefully performed at day five after ovulation with a nonsurgical trans-cervical procedure, using a standard artificial insemination pipette, protected with a sterile outer chemise. Conceptus fragments (2 mL) were deposited into the uterine body.

### Uterine fluid samples

Uterine fluid samples were collected using commercial vaginal tampons (Mini OB; Johnson & Johnson Industrial Ltd, São José dos Campos, São Paulo, Brazil). Tampons were aseptically introduced into the uterus, according to (Reilas, 2001), modified by (Malschitzky et al., 2008). The tampon was inserted through the cervix, protected by a palpation glove (double-glove technique). The distal part of a palpation glove was cut to form a plastic tube, and the gloved hand with the tampon was introduced in the plastic tube, closing its end with a finger. The plastic tube was removed at the moment of tampon's introduction in the uterus, where it remained for 30 min. The tampon was then removed, protected by a palpation glove, and immediately inserted in a sterile plastic sack and centrifuged (1500 x g, for 10 min) for fluid recovery. The recovered fluid (> 0.5 mL) was transferred to conic tubes and immediately centrifuged at 4 °C (10.000 x g, for one h). The supernatant was transferred to cryovials in 500 μL aliquots and stored at -80 °C until further analyses.

### Electrophoresis

Protein content from uterine samples was determined using the Bradford method (Bradford, 1976), using 1 mg/mL of BSA (A7906; Sigma-Aldrich, St. Louis, MO, USA) as standard. Proteins were separated using two-dimensional gel electrophoresis in duplicate. In summary, samples containing 250 µg of total protein were mixed with buffer (7-M urea, 2-M thiourea), 0,5% free ampholytes (IPG buffer, pH 3-10 [GE Life Sciences, Piscataway, NJ, USA]), 2% dithiothreitol (DTT), 2% CHAPS, and traces of bromophenol blue. Initially, strips were rehydrated for 16 h in 250 µL rehydration solution at room temperature in IPG Box (GE Life Sciences). Samples were incubated in 13 cm IPG strips (pH 3 - 10 linear, GE Life Sciences). Isoelectric focusing was carried out in Ettan IPGphor III System (GE Life Sciences) with the following conditions: 100 V (100 V/h), 150 V (75 V/h), 200 V (200 V/h), 500 V (500 V/h), 1000 V (800 V/h), 8000 V (11300 V/h), 8000 V (14412 V/h), 8000 V (7900 V/h), by a total of 35000 V. Current limit was 50 mA per strip. Finished isoelectric focalization, strips were stored to -80 °C.

For the second dimension, strips containing endometrial proteins were thawed at room temperature and incubated for 15 min in equilibration buffer I (75 mM Tris-HCl, pH 8.8, 6-M urea, 29.3% glycerol, 2% SDS – sodium dodecyl sulfate, 2% DTT) and equilibrated for an additional 15 min in buffer II (similar to buffer I, but containing 2.5% iodoacetamide instead of DTT). Subsequently, strips were placed on top of 1.5 mm thick 12.5% SDS polyacrylamide gels and fixed with agarose sealing solution (25 mM Tris base, 192 mM glycine, 0.1% SDS, 0.5% agarose, 0.002% bromophenol blue). Proteins were separated using SE 600 Ruby system (GE Life Science) at 20 °C. Electrophoresis was performed with 15 mA for 15 min per plate starting at 90 V, followed by 40 mA per plate at 250 V for 4 h with 30 Watts (Electrophoresis Power Supply 301; Amersham Pharmacia Biotech).

Gels were stained in colloidal Coomassie blue (Kang et al., 2002) with modifications (Dyballa and Metzger, 2009). Briefly, gels were washed three times (20 min each) with Milli-Q water. The gels were placed in a solution with phosphoric acid 2% (85%), ethanol (10%), ammonium sulfate (5%), and Coomassie Blue G-250 solution (0.02%), in agitation on a shaker for 24 h. Later, gels were washed three times (20 min each) with Milli-Q water and placed in a destaining solution (phosphoric acid 2% (70%) and ethanol (30%) for 12 h and stored in acetic acid 5%.

Two-dimensional gels were scanned using ImageScanner III (GE Life Sciences) at 300 dpi and analyzed to determine the relative volume of each spot considering the volume over all the spots in the image using software ImageMaster™ 2D Platinum (version 7.0; GE Life Sciences). Proteins, in critical regions, were used as landmarks, and final spot matches were organized by checking each spot in each gel with a particular pattern.

### Spot selection criteria

Spots were selected by (a) presence in at least 80% of gels in one of the groups (Cyclic or Fragment), (b) significant abundance (P ≤ 0.01) of relative volume in one of the groups (Cyclic or Fragment), and (c) a minimum of 1.5-fold magnitude difference between groups using ImageMaster™ 2D Platinum (version 7.0; GE Life Sciences).

### In-gel tryptic digestion and mass spectrometry

Spots were manually excised, and digested using Shevchenko et al. (2006) protocol Tryptic peptides were dried, by vacuum centrifugation (Eppendorf, Germany), and samples were re-suspended in 10 μL of 0.1% trifluoroacetic acid solution and desalted in Zip Tip, model ZTC18S096 (Millipore, USA) and eluted in 3 μL of 50% acetonitrile, acidified with 0.1% trifluoroacetic acid. An eluted peptide solution (1 mL) was placed in the matrix of αcyano-4-hydroxycinnamic acid-HCCA (Bruker Daltonics, Germany), solubilized in the same solution from which tryptic peptides were eluted, to a final concentration of 10 mg/mL. Calibration of the analysis method MS1 was performed placing in the matrix peptides mix (Peptide Calibration Standard II) (Bruker Daltonics, Germany).

#### Protein identification using MALDI-TOF/TOF

The spectra of MS1 and MS2 were acquired in a MALDI-TOF/TOF spectrometer, Ultraflex III model (Bruker Daltonics). MS1 data were obtained with positive ionization and reflector mode, with a detection range of 500 - 3400 Da. For the MS2, using the positive ionization and LIFT technique, the ratio mass/charge (m/z) of the ions with the highest intensity was selected.

All data obtained were managed by Flexcontrol software, version 3.3 (Bruker Daltonics, Germany). The spectra resulting from the MS1 and MS2 analyzes were processed using the FlexAnalysis software, version 3.3 (Bruker Daltonics, Germany). The peak lists of MS2 were generated in the Mascot generic format by the BioTools application, version 3.2 (Bruker Daltonics, Germany).

#### Protein identification using NanoUHPLC-QTOF

The tryptic peptides from the excisions were solubilized in 70 μL of 0.1% formic acid solution (v/v). The peptides were analyzed using Liquid Chromatography-Mass Spectrometry (LC-MS) using the nanoACQUITY UPLC system (Waters, Milford, MA, USA) and a mass spectrometer micrOTOF QII® (Bruker Daltonics, Bremen, Germany), with a microESI ionization needle. Chromatographic analysis of the samples was executed in a trap column and a capillary column ProteCol GHQ303 C18 3.0 μm–300 μm × 150 mm, operating at a flow rate of 4.5 μL min, using mobile phase solutions.

Mobile phase solutions were carried with the following solutions: water and 0.1% formic acid (v/v) (A) and acetonitrile and 0.1% formic acid (v/v) (B). Gradient program started in B at 5% by 14 min, a linear rising ramp of B at 5% for 50% in 30 min, maintaining in B at 50% by 5 min, linear rising ramp of B at 50% for 90% in 3 min, maintaining of B at 90% by 2 min, linear gradient descent of B at 90% for 10% in 3 min, and maintaining of B at 10% by 3 min. The acquisition of data lasted approximately 60 min. The scanning of ions for MS1 spectra in positive mode was carried out for masses ranging between 100 and 2000 m/z, and between 70 and 2000 m/z for the MS2 spectra. The mass spectrometer micrOTOF QII® analysis was realized by the Hystar software program, version 3.2 (Bruker Daltonics, Bremen, Germany), and the spectra were processed through the Data Analysis software program, version 4.0 (Bruker Daltonics, Bremen, Germany). The mass list was generated in the format extensible mark-up language (*. mzXML).

### Protein identification and characterization

The mass lists were compared with protein sequences of the Equidae family database, available at the UniProt Knowledgebase (UniProtKB, 2018; downloaded on 13/12/2018, with 29,702 entries), using the Mascot Daemon software, version 2.4.0 (Matrix Science, London, UK). Mascot search parameters were (a) enzymatic digestion by trypsin with one missed cleavage (allowing an error tolerance of 0.2 Da for the parental ion and 0.5 Da for the fragments), (b) carbamidomethylation of cysteine as fixed modification and (c) oxidation of methionine as a variable modification.

The mass list in format mzXML was analyzed using software PEAKS version 7.0 (Bioinformatics Solutions Inc., Canada) (Ma et al., 2003). The following parameters were used in the search: enzymatic digestion by trypsin with one missed cleavage, carbamidomethylation of cysteine as a fixed modification, and oxidation of methionine as a variable modification; error tolerance was of 30 ppm MS/MS for the parental ion and 0.02 Da for the fragments. Proteins were considered identified when these presented at least one unique peptide with FDR (False Discovery Rate) < 1%.

The Scaffold software, version 3.6.4 (Proteome Software Inc., Portland, OR), was used to validate Mascot results by applying Peptide Prophet (Keller et al., 2002) and Protein Prophet (Nesvizhskii et al., 2003) algorithms. Proteins and peptides were statistically validated when the probability of identity was equal or above 90%. Estimation was performed based on a complete amino acid sequence of protein deposited in the selected database as a reference for the identification.

Useful information about identified proteins was retrieved from UniProt Knowledgebase (UniProt Consortium, 2015). Protein sequences were also functionally annotated using Blast2GO version 5.2.5 (Götz et al., 2008). In this analysis, proteins were aligned with protein database sequences (NCBI, 2018) using BLASTp of Blast version 2.8.1 (Altschul et al., 1990) (E-value threshold 1e-25). Comparing their sequences with the Eukaryotic Orthologous Groups of proteins database (KOG) identified and statistically validated proteins were classified (Koonin et al., 2004). Using the Reverse Position Specific BLAST (RPS-BLAST), a KOG ID was assigned to each protein that significantly aligned (E-value threshold 1E-25) with those deposited in the database.

Theoretical molecular weight (MW) and isoelectric point (pI) for each protein were estimated using the ProtParam tool (SIB, 2018). Estimation was performed based on a complete amino acid sequence of the protein deposited in the selected database as a reference for identification.

The interaction networks between the identified and validated proteins were reconstructed using the software STRING (Search Tool for the Retrieval of Interacting Genes) version 10.0 (String Consortium, 2018; Szklarczyk et al., 2015).

### Statistical analysis

The relative volume of each spot (optical density x area) was normalized to the total volume of spots detected on each gel for comparison group. Software ImageMaster™ 2D Platinum (version 7.0; GE Life Sciences) was used to detect differential abundance. Spots data were submitted to ANOVA to evaluate the relative volume of each protein spot as a dependent factor, and the Groups (Cyclic or Fragment) were considered as independent variables using the software GraphPad Prism (Version 8.4.2). False discovery rate was calculated and corrected with a significance level by Benjamini-Hochberg corresponding to P < 0.05 was q* = 0.013.

## Results

The protein concentration recovered from uterus showed a median of 2.36 ± 0.21 μg/μL for the Cyclic group and 2.5 ± 0.3 μg/μL for the Fragment group, without differences between groups (P = 0.255).

**Figure 1:**
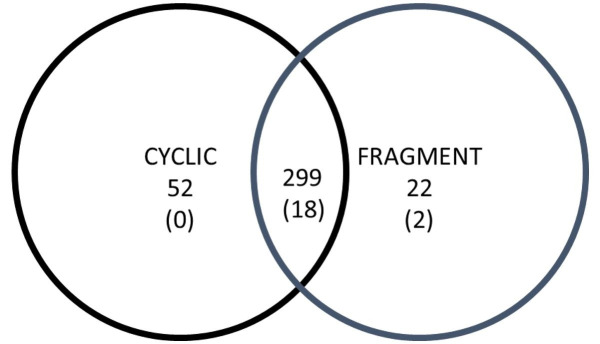
Venn diagram depicting matches between the Cyclic and Fragment group. The number of detected spots are presented without parentheses and the number of identified spots in parentheses.

**Figure 2:**
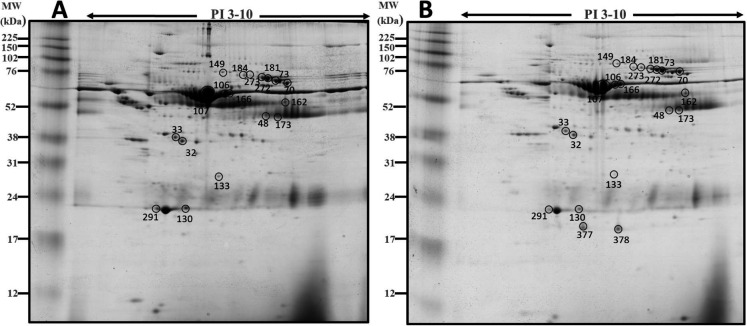
Representative 2D PAGE gel with spots from the uterine fluid of (A) Cyclic (18 spots) and (B) Fragment (20 spots) groups. The numbers in the figure correspond to the Spot ID shown in Tables 1 and 2.

A total of 373 spots were detected. MW ranged from 12 to 225 kDa and pI from 3 to 10. The spots mean number observed in gels of the Fragment group (280 ± 30) did not differ (P = 0.763) with the mean number of the Cyclic group (291 ± 25). Total matches in the two groups were 299 spots (Figure 1). Spots identified only in gels of Cyclic and Fragment groups were 52 and 22, respectively. A total of 28 spots satisfied selection criteria, being two presents exclusively in the Fragment group. Eight spots presented low intensity and were discarded, and 20 spots were carried for identification (Figure 2).

MASCOT and PEAKS software identified 13 proteins in the 20 spots with more abundance in the Cyclic (Table 1) and Fragment (Table 2) groups. Five proteins were identified as “Uncharacterized,” and three of these proteins presented the same access number (48, 162, and 173) (Table 1 and 2). Protein functional classification generated by the software Blast2GO is listed in Table 3.

**Table 1:** Proteins identified with higher abundance in the uterine fluid of the Cyclic group using MALDI TOF/TOF and LC-MS/MS, with MASCOT and PEAKS software, and compared with Database Equidae / Uniprot.

**Spot** **a**	**Protein name**	**GENEb**	**Access number**	**Protein scorec**	**Coveraged**	**Theoretical/Experimental**		**Peptide sequence**	**Peptide scoree**
						**MW (kDa)**	**pI**		
70	Uncharacterized protein	TF	F6ZEH8	156.12#	11%	109.1/71.0	8.39/8.0	TEPQTHYYAVAVVK (*)	36.38≈
								SKDFHLFSSPHGK (*)	42.65≈
								HCEFDKFFR (*)	52.56≈
								EGCAPGYR (*)	32.74≈
								YYGYTGAFR (**)	97%‡
				88Ϯ				CLVEKGDVAFVK (*)	32.75≈
								SGNFQLLCPDGTRK (*)Ϯ	31.75≈
								AVTEFESCNLAK (*)Ϯ	55.38≈
								APNHAVVSR (*)	44.63≈
								YLTAVANLR (*)	46.49≈
106	Serum albumin	ALB	F7BAY6	226.52#	39%	68.3/61.0	5.78/6.59	SEIAHR (*)	43.87≈
								FNDLGEK (*)	44.74≈
								LVNEVTEFAKK (*)	44.90≈
								SLHTLFGDK (*)	43.47≈
								LCTVATLR (*)	27.11≈
								DDHPNLPK (*)	26.39≈
				336Ϯ				YLYEVAR (***)	54.18≈
								ADFTECCPADDKAGCLIPK (*)	43.22≈
								CSSFQNFGER (*)	71.73≈
								ECCHGDLLECADDRADLAK (*)	42.15≈
								YICEHQDSISGK (*)	62.78≈
								DVFLGTFLYEYSR (**)	95%‡
								RHPDYSVSLLLR (***)	41.42≈
								IAKTYEATLEK (*)	44.51≈
								KAPQVSTPTLVEIGR (*)	65.06≈
								LPESER (*)	43.82≈
								LPCSENHLALALNR (*)	63.71≈
								LCVLHEK (*)	34.36≈
								TPVSEK (*)	49.51≈
								RPCFSALELDEGYVPK (***)	53.01≈
								KQSALAELVK (*)	38.35≈
								TVLGNFSAFVAK (*)	45.67≈
130	Immunoglobulin lambda light chain variable region	IGL	A0A0A1E6K7	87.25#	11%	23.1/23.0	7.61/5.8	VNDAVTTDGVQTTR (*)	65.93≈
								SYSSVSCQVK (*)	27.03≈
	Apolipoprotein A1	APOA1	F6Z2L5	234Ϯ	11%	30.3/23.0	5.65/5.8	VAPLSDEFR (**)	99%‡
								VNLAPFSEELR (**)	99%‡
								AHPALEDLR (**)	97%‡
133	Immunoglobulin lambda light chain variable region	IGL	A0A0A1E6K7	84.36#	10%	23.1/29.0	7.61/6.5	VNDAVTTDGVQTTR (*)	54.17≈
								YAASSYLTR (*)	47.38≈
162	Uncharacterized protein	IGHCp*	H9GZT5	76.96#	7%	36.4/56.0	8.27/8.13	VVSILAIQHK (*)	41.71≈
								ALPAPVER (*)	31.20≈
								LTVETNR (*)	35.92≈
166	Hemopexin	HPX	F6X1I8	101.93#	7%	51.3/62.0	7.58/6.7	NFIGPADAAFR (*)	43.79≈
								FNPVSGEVPPK (*)	41.65≈
								GGHTLVDGYPK (*)	56.53≈
291	Apolipoprotein A1	APOA1	F6Z2L5	130.67#	18%	30.3/23.0	5.65/5.12	EYVAQFEASALGK (*)	59.21≈
								DTEGLRQELNK (*)	43.92≈
				70Ϯ				VAPLSDEFREGAR (*)	42.98≈
								VNLAPFSEELR (**)	99%‡

a Spot ID corresponds to the numbers shown in Figure 2; ^b^Gene designation in UniProt. N/A Gene not characterized; ^c^Score Protein of PEAKS(#) or MASCOT (Ϯ); ^d^Protein coverage calculated (identified amino acids/total amino acids); ^e^Peptide score data obtain of PEAKS (P-value of the probability of correspondence ≈) or SCAFFOLD (sequence probability percentage ‡). MW – Molecular Weight; pI - Isoelectric point. (*) Acquisition of mass spectra of the peptide identified by LC-MS / MS; (**) Acquisition of mass spectra of the peptide identified by MALDI TOF/TOF; (***) Acquisition of mass spectra of the peptide identified by both LC-MS/MS and MALDI TOF/TOF.

**Table 2:** Proteins identified with higher abundance in the uterine fluid of the Fragment group using MALDI TOF/TOF and LC-MS/MS, with MASCOT and PEAKS software, and compared with Database Equidae / Uniprot.

**Spot** **a**	**Protein name**	**GENEb**	**Access number**	**Protein scorec**	**Coveraged**	**Theoretical/Experimental**		**Peptide sequence**	**Peptide scoree**
						**MW (kDa)**	**pI**		
32	Leukocyte elastase inhibitor	SERPINB1	P05619	79.39#	16%	43.0 /39	5.78/5.68	ADLSGMSGAR (*)	54.12≈
								LGVQDLFNR (*)	50.55≈
								VLELPYQGK (*)	48.81≈
				196Ϯ				IPELLVK(*)	34.25≈
								ALYFDTVEDIHSR (**)	98%‡
								HNPSANILFLGR (**)	100%‡
33	Fibrinogen beta chain	FGB	F6PH38	71.57#	10%	55.7/40.0	8.63/5.5	ALYEGFTVK (***)	41.50≈
								QGFGNIATNADGK (*)	28.89≈
				185Ϯ				IRPYFPQQ (*)	27.64≈
								QDGSVDFGR (**)	92%‡
								EDGGGWWYNR (**)	97%‡
48	Uncharacterized protein	IGHCp*	H9GZT5	91.65#	19%	36.4/47.0	8.27/7.5	VVSILAIQHK (*)	47.13≈
								DVLMISR (*)	37.62≈
								SQTYICNVAHPASSTK (*)	29.70≈
				129Ϯ				VSVTCLVK (*)	28.74≈
								LTVETNR (*)	27.43≈
								EPQVYVLAPHRDELSK (**)	99%‡
	Immunoglobulin gamma 1 heavy chain constant region	IGHC1	Q95M34	97.46#	12%	37.4/47.0	7.68/7.5	FNWYMDGVEVR (*)	55.97≈
								IQHQDWLSGK (*)	39.67≈
								VNNQALPQPIER (*)	42.14≈
								VSVTCLVK (*)	28.74≈
73	Serotransferrrin	TF	P27425	211.04#	24%	78.0/71.0	6.83/7.7	SIVPAPPLVACVK (*)	55.82≈
								RTSYLECIK (*)	38.76≈
								KNSNFQLNQLQGK (*)	44.23≈
								CLADGAGDVAFVK (*)	70.04≈
								SKDFHLFSSPHGK (*)	54.06≈
								DSALGFLR (*)	45.89≈
								SSSDPDLTWNSLK (*)	55.81≈
								HCEFDKFFR (*)	55.43≈
				76Ϯ					
								YYGYTGAFR (***)	51.49≈
								CLVEKGDVAFVK (*)	59.20≈
								HQTVEQNTDGRNPDDWAK (*)	54.48≈
								SCYLAR (*)Ϯ	38.27≈
								AACVCQELHNQQASYGK (*)Ϯ	57.02≈
								YLTAVANLR (*)	52.77≈
								LLEACTFHRV (*)Ϯ	50.24≈
107	Serum albumin	ALB	F7BAY6	247.80#	50%	68.3/58.0	5.78/6.3	DTHKSEIAHR (*)	53.72≈
								FNDLGEK (*)	25.96≈
								LVNEVTEFAKK (*)	38.01≈
								CAADESAENCDK (*)	66.75≈
								SLHTLFGDKLCTVATLR (*)	61.95≈
								ATYGELADCCEK (*)	48.10≈
								DDHPNLPK (*)	46.72≈
								ADFTECCPADDKAGCLIPK (*)	31.66≈
								LDALKER (*)	34.17≈
								LSQKFPK (*)	39.65≈
								ADFAEVSKIVTDLTK (*)	47.17≈
								ECCHGDLLECADDRADLAK (*)	28.69≈
								YICEHQDSISGK (*)	43.12≈
								ACCDKPLLQK (*)	43.84≈
								SHCIAEVK (*)	43.75≈
								DAKDVFLGTFLYEYSR (*)	42.59≈
								RHPDYSVSLLLR (***)	58.66≈
								IAKTYEATLEK (*)	41.89≈
								KAPQVSTPTLVEIGR (*)	66.02≈
								TLGKVGSR (*)	42.99≈
								LPESERLPCSENHLALALNR (*)	48.49≈
								LCVLHEKTPVSEK (*)	54.06≈
								CCTDSLAER (*)	43.70≈
								RPCFSALELDEGYVPK (***)	48.74≈
								KQSALAELVK (*)	34.81≈
								ATKEQLK (*)	36.04≈
								TVLGNFSAFVAK (*)	60.22≈
								CCGAEDKEACFAEEGPK (*)	60.69≈
								LVASSQLALA (*)	38.07≈
149	Uncharacterized protein	CFB	F6RMD0	76.57#	3%	85.9/89.0	6.75/7.03	LEDSVTYYCSR (*)	30.83≈
								DISAVVTPR (*)	61.15≈
173	Uncharacterized protein	IGHCp*	H9GZT5	154.02#	24%	36.4/47.0	8.27/7.79	SQTYICNVAHPASSTK (*)	69.58≈
								DVLMISR (*)	44.61≈
								VVSILAIQHK (*)	57.55≈
								TISKPTGQPR (*)	58.01≈
				67Ϯ				EPQVYVLAPHR (**)	95%‡
								VSVTCLVK (*)	46.80≈
								DFYPTDIDIEWK (*)	43.58≈
								LTVETNR (*)	40.49≈
	Immunoglobulin gamma 1 heavy chain constant region	IGHC1	Q95M34	97.01#	11%	37.4/47.0	7.68/7.79	IQHQDWLSGK (*)	53.81≈
								SQEPQVYVLAPHPDELSK (*)	42.66≈
								VSVTCLVK (*)	46.80≈
181	Serotransferrin	TF	P27425	178.76#	11%	78.0/72.0	6.83/7.62	RTSYLECIK (*)	33.82≈
								TEPQTHYYAVAVVK (*)	70.57≈
								SKDFHLFSSPHGK (*)	52.55≈
								YYGYTGAFR (***)	55.17≈
				147Ϯ				SCYLAR (*)Ϯ	39.43≈
								APNHAVVSR (*)	47.29≈
								YLTAVANLR (*)	40.12≈
								LLEACTFHR (*)Ϯ	48.50≈
184	Fibrinogen gamma chain	FGG	F6W2Y1	241Ϯ	8%	49.7/75.0	5.41/7.16	YEALVVTHESTIR (**)	99%‡
								IHLISTQTTIPYVLR (**)	99%‡
								VGPENDKYR (**)	98%‡
272	Serotransferrin	TF	P27425	118Ϯ	3%	78.0/73.0	6.83/7.7	YYGYTGAFR (**)	93%‡
								APNHAVVSR (**)	92%‡
273	Fibrinogen gamma chain	FGG	F6W2Y1	124.40#	12%	49.7/74.0	5.41/7.49	YEALVVTHESTIR (*)	74.60≈
								VAQLEAK (*)	34.80≈
								LDGSVDFKK (*)	32.32≈
								IHLISTQTTIPYVLR (**)	96% ‡
								VQLEDWNGK (*)	49.87≈
	Serotransferrin	TF	P27425	70.76#	2%	78.0/74.0	6.83/7.49	DSALGFLR (*)	35.62≈
								LLEACTFHR (*)	47.13≈
377	Lipocalin 2	LCN2	F6TIR2	280Ϯ	17%	23.1/20.0	5.94/5.8	LIPAPPLDR (**)	98%‡
								YFGVQSYIVR (**)	100% ‡
								VADTDYNQFAIVFFR (**)	100%‡
378	Lipocalin 2	LCN2	F6TIR2	154.20#	44%	23.1/20.0	5.94/6.65	LIPAPPLDR (**)	99%‡
								VPLQPDFKDDQFQGK (*)	57.29≈
								KEEQGQFTMYTTTYELK (*)	48.29≈
								DQNCDHWIR (**)	97%‡
				326Ϯ				YFGVQSYIVR (***)	63.22≈
								NQEYFKTTLYR (*)	41.76≈
								TKELTPELR (**)	94%‡
								EKFISFAK (***)	41.10≈

a Spot ID corresponds to the numbers shown in Figure 2; ^b^Gene designation in UniProt or Abbreviation. *Abbreviation of the protein without gene identification; ^c^Score Protein of PEAKS(#) or MASCOT (Ϯ); ^d^Protein coverage calculated (identified amino acids/total amino acids); ^e^Peptide score data obtain of PEAKS (P-value of the probability of correspondence ≈) or SCAFFOLD (sequence probability percentage ‡). MW – Molecular Weight; pI - Isoelectric point. (*) Acquisition of mass spectra of the peptide identified by LC-MS / MS; (**) Acquisition of mass spectra of the peptide identified by MALDI TOF/TOF; (***) Acquisition of mass spectra of the peptide identified by both LC-MS/MS and MALDI TOF/TOF.

**Table 3:** Functional classification of the proteins identified in the uterine fluid of Cyclic and Fragment mares by Blast2GO with “Uncharacterized protein.”

**ID Spots**	**GENE / ABV** **a**	**UniProtKB IDb**	**Lengthc**	**Description**	**e-Valued**	**Similarity mean (%)e**	**GOsf**
106, 107	ALB	F7BAY6	607	Serum albumin	0	90.86	17
32	SERPINB1	P05619	379	Leukocyte elastase inhibitor	0	92.74	4
33	FGB	F6PH38	490	Fibrinogen beta chain	0	92.19	29
48, 162, 173	IGHCp*	H9GZT5	335	Immunoglobulin gamma heavy chain precursor	0	83.9	0
48, 173	IGHC1	Q95M34	337	Immunoglobulin gamma 1 heavy chain constant region	0	84.41	9
70	TF	F6ZEH8	990	Serotransferrin-like	0	84.99	8
73, 181, 272, 273	TF	P27425	706	Serotransferrin	0	89.09	7
130, 133	IGL	A0A0A1E6K7	221	Immunoglobulin lambda light chain variable region	1,78E-159	94.97	0
130, 291	APOA1	F6Z2L5	266	Apolipoprotein A-I	0	90.28	59
149	CFB	F6RMD0	768	Complement factor B	0	93.08	7
166	HX	F6X1I8	462	Hemopexin	0	88.88	14
184, 273	FGG	F6W2Y1	437	Fibrinogen gamma chain	0	93.36	21
377, 378	LCN2	F6TIR2	199	Neutrophil gelatinase-associated lipocalin	2,89E-147	90.14	1

a Gene or Abbreviation. *Abbreviation of the protein without gene identification; ^b^Identification in the UniProtKB database; ^c^Protein Length; ^d^E-Value: the number of alignments expected by chance (The lower the E value, the more significant the score, and the alignment); ^e^Probability Similarity means (%) for validation by Blast2GO software of the proteins statistically; ^f^number of Gene Ontology.

**Figure 3:**
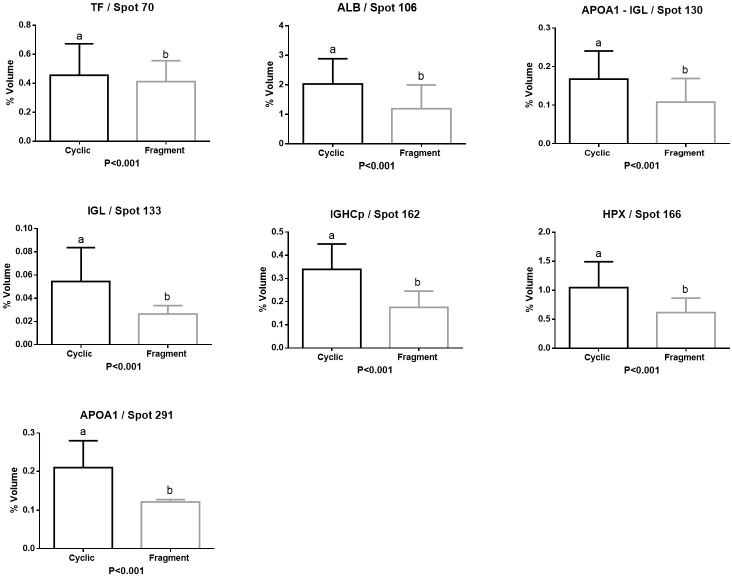
Relative volume (%) of proteins with higher abundance in the uterine fluid of the Cyclic group. Different letters (a,b) represent significant differences (P < 0.01).

Proteins with more abundance in Cyclic group were Serotransferrin-like (TF/ Spots 70), Serum albumin (ALB/ Spots 106), Immunoglobulin lambda light chain variable region (IGL/ Spot 130, 133), Apolipoprotein A1 (APOA1/ Spots 130, 291), Immunoglobulin Gamma heavy chain precursor (IGHCp/ Spot 162), and Hemopexin (HPX/ Spot 166) (Figure 3).

**Figure 4:**
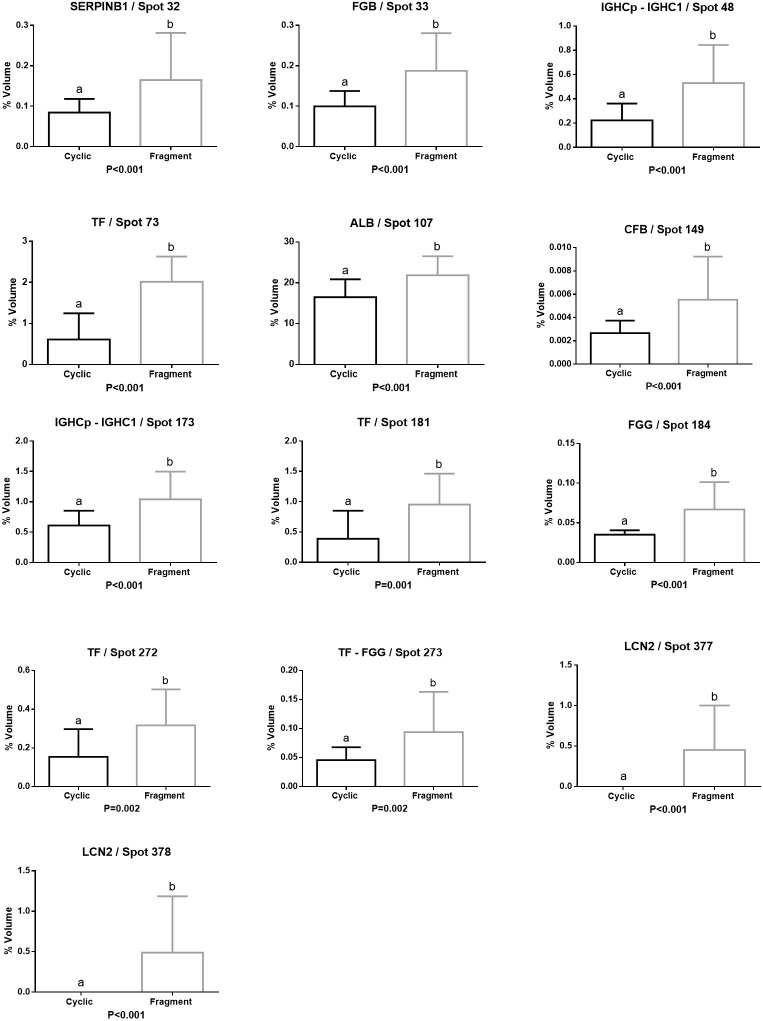
Relative volume (%) of proteins with higher abundance in the uterine fluid of the Fragment group. Different letters (a,b) represent significant differences (P < 0.01).

Leukocyte elastase inhibitor (SERPINB1/ Spot 32), Fibrinogen beta chain (FGB/ Spot 33), Immunoglobulin gamma heavy chain precursor (IGHCp/ Spots 48, 173), Immunoglobulin gamma one heavy chain constant region (IGHC1/ Spots 48, 173), Serotransferrin (TF/ Spots 73, 181, 272, 273), Serum albumin (ALB/ Spots 107), Complement factor B (CFB Spot 149) and Fibrinogen gamma chain (FGG/ Spots 184, 273), were the most abundant proteins in Fragment group. Lipocalin 2 (LCN2/ Spots 377, 378) was only identified in the Fragment group (Figure 4).

Two proteins, ALB and TF, showed higher abundance in the Cyclic and Fragment groups. In four spots were identified two proteins (48-173 IGHCp, IGHC1; 130 IGL, APOA1; 273 FGG, TF) (Table 1 and 2).

**Figure 5:**
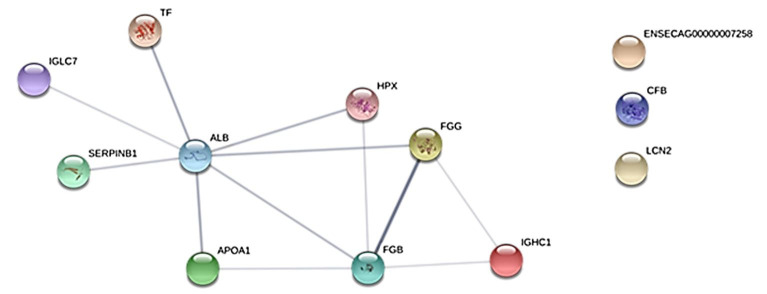
Protein-protein interactions (PPI) network via STRING 10. Interactions of proteins that differed significantly in their abundance. IGLC7 is the identification of IGL, and ENSECAG00000007258 is the identification of IGHCp for STRING.

Table 4 shows the functional classification of statistically validated proteins based on the KOG Database. Figure 5 depicted analysis of protein-protein interactions (PPI) network via STRING 10.

**Table 4:** Functional classification of proteins identified in the uterine fluid of Cyclic and Fragment mares by KOG Database.

**Spot**	**GENE**	**UniProtKB ID**	**KOG ID**	**KOG Group**	**Description**	**KOG class**	**e-Value**
32	SERPINB1	P05619	KOG2392	V	Serpin	Defense mechanisms	7,66E-146
33	FGB	F6PH38	KOG2579	R	Ficolin and related extracellular proteins	General function prediction only	9,55E-117
70	TF	F6ZEH8	KOG0090	U	Signal recognition particle receptor, beta subunit (small G protein superfamily)	Intracellular trafficking, secretion, and vesicular transport	2,04E-96
149	CFB	F6RMD0	KOG3627	E	Trypsin	Amino acid transport and metabolism	8,52E-30
166	HX	F6X1I8	KOG1565	O	Gelatinase A and related matrix metalloproteases	Posttranslational modification, protein turnover, chaperones, Extracellular structures	1,03E-60
273	FGG	F6W2Y1	KOG2579	R	Ficolin and related extracellular proteins	General function prediction only	4,30E-109

## Discussion

The present study focused on verifying protein abundance differences, seven days after ovulation, between uterine fluid of cyclic mares and infused mares with conceptus fragments. Many transcripts and proteins identified previously in early equine pregnancy showed a great diversity of regulatory mechanisms that underlie early development (Pillai et al., 2018). This study used an experimental design expecting to identify relevant proteins in embryo-maternal communication. A large number of spots were observed in both groups, indicating a considerable diversity of proteins and probably a range of post-translational changes.

Thirteen-day-old concepti were selected due to higher content of proteins in the yolk sac (Smits et al., 2018), with one or more of these proteins probably involved in the anti-luteolytic mechanism (McDowell et al., 1990) which prolongs the lifespan of corpus luteum during maternal recognition of pregnancy (Sharp et al., 1989). Proteins present in conceptus tissues remain inactive with unfolded conformations during storage in low temperatures, resulting in active molecules after the thawing process (Garber-Cohen et al., 2010). Days of fragments infusion (Day 5) and uterine fluid sample collection (Day 7) were selected because it is the period where serum progesterone concentration reaches its peak both in pregnant and cyclic mares (Camozzato et al., 2019).

**Figure 6:**
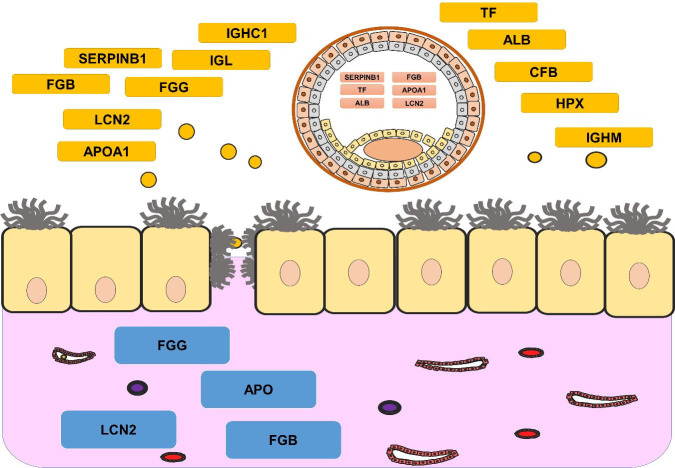
Schematization of the uterine interaction with proteins and transcripts identified, based on current experiments [1,3,6,10,12,33,35]. Proteins in the red box are proteins detected into the embryo, proteins in the yellow box are proteins detected in the uterine fluid, and proteins in the blue box are proteins detected in the endometrium.

Infusion with conceptus fragments showed endometrial and vascular changes associated with a protein stimulus (Camacho et al., 2018), suggesting similarity with early equine pregnancy process (Caballeros et al., 2019; Camozzato et al., 2019). Proteins, reported in the present research, were identified in current studies of proteins and transcripts of endometrial tissues, embryonic tissues, and uterine fluid (Klein et al., 2010; Swegen et al., 2017; Maloney et al., 2018; Smits et al., 2018; Bastos et al., 2019) (Figure 6).

Some proteins (LCN2, CFB, and IGHCp) identified in the present study showed an independent role in the protein-protein interaction network obtained from the STRING v10 database.

LCN2 was detected only in the Fragment group. This protein has been identified in previous studies in the yolk sac and increasing its expression in the uterine fluid of early pregnant mares compared with cyclic ones (Hayes et al., 2012; Haneda et al., 2017 ;Smits et al., 2018). LCN2 is known as a multifunctional protein, playing a role in homeostasis and iron transport (Miyamoto et al., 2011). Interacting with interleukins LCN2 shows an iron delivery pathway crucial to survival, growth, and maturation of cells that can carry to apoptosis regulation (Devireddy et al., 2001; Lin et al., 2011). LCN2 also has been correlated in processes of embryogenesis (Zhang et al., 2012) and, suggested as a mediator of innate immunity in the reproductive process by stimulation of cytokines (Tadesse et al., 2011). Studies in women suggest that LCN2 plays an essential role in the invasion of extravillous trophoblast in early placentation under hypoxia by matrix metalloproteinase-9 pathway stimuli (Kobara et al., 2013).

CFB was another protein identified in the present study that shows an independent role. The CFB gene is localized in the major histocompatibility complex and belongs to the C3-convertase complex. In human has been suggested that the oviduct possesses C3-convertase and in the presence of preimplantation embryos may be involved in the production of embryotrophic inactivated complement-3b (iC3b), which stimulates blastocyst development (Tse et al., 2008). In early pregnancies of cows and ewes, a decrease of CFB was detected, likely indicating that the protein is being used by the embryos to generate iC3b (Koch et al., 2010; Muñoz et al., 2011). In the present study, CFB abundance increased in the Fragment group, probably the proteins of conceptus fragments signalized the production of CFB, but in the absence of a “viable embryo,” the protein increased in uterine lumen, without modifications. Dysregulation of CFB has been suggested in pregnant women with preeclampsia (Lynch et al., 2016).

Several immunoglobulins were identified, in the present study, with different MW and pI: IGL (2), IGHCp (3), and IGHC1 (2). IGL is more abundant in the Cyclic group than in the Fragment group. IGHCp presented higher abundance in both Cyclic and Fragment groups. When the higher abundance occurred in the Fragment group, IGHCp shared the spot with IGHC1. This rise of IGs can be associated with an increase in immune cells reported after the infusion of conceptus fragments (Camacho et al., 2018). The higher abundance of IGs in the uterine fluid has been detected in pregnant mares compared with cyclic mares (Hayes et al., 2012; Smits et al., 2018). IGs presence could probably explain the activation of the maternal immune system with conceptus fragments (Hansen, 2011) or anti-microbial protection for the “pregnancy” (Parr and Parr, 1985).

IGHC1 showed connection with Fibrinogens (FG) units in the protein-protein interaction network. A higher functional connection was identified between FG Alpha and Gamma. These proteins showed a greater abundance in the Fragment group than in the Cyclic group. In early equine pregnancy, FG proteins have been detected in embryo capsule (Klein et al., 2010), blastocoel (Swegen et al., 2017), intrauterine secretion (Smits et al., 2018; Bastos et al., 2019) and conceptus' yolk-sac (Smits et al., 2018). FG probably promote cessation of conceptus mobility and contributes to conceptus fixation via binding to endometrial integrins (Klein, 2016). In mice, it has been documented that FG subunits play a critical role in the maintenance of pregnancy, supporting proper development of fetal-maternal vascular communication and stabilization of embryo implantation; moreover, it aids the correct formation of yolk sac (Iwaki et al., 2002). This abundance of FG subunits in the Fragment group may have a relation with the increase of vessel diameter and vascular endometrial changes previously reported after the infusion of conceptus fragments (Camacho et al., 2018).

FG also showed a functional connection with HPX and APOA1. This protein has been identified in the uterine fluid of humans (DeSouza et al., 2005; Al-Rumaih et al., 2006) and equine (Hayes et al., 2012; Maloney et al., 2018; Smits et al., 2018). Increment of HPX has been detected with a higher presence of E2 (DeSouza et al., 2005) and in blood during pregnancy (Chen and Khalil, 2017), showing an association of HPX with matrix metalloproteinases-9 by dimerization patterns promoting vascular remodeling (Chen et al., 2017). Interestedly, the vessel diameter and vascular index by Doppler increased after the infusion of conceptus fragments (Camacho et al., 2018). HPX in the present study showed a higher abundance in the Cyclic group suggesting that HPX may be conjugated in blood vessels, and probably these vascular changes are mediated with the aid of the FGB.

Transcripts of apolipoproteins as APOA1 have been previously reported in equine conceptus and endometrium (Klein and Troedsson, 2011b). APOA1 has been identified in equine conceptus and uterine fluid (Swegen et al., 2017; Smits et al., 2018), suggesting that it is likely involved in guarantee the nutritional demands of the embryo, ensuring adequate transport of lipids by endocytosis pathway (Smits et al., 2018). In human, it has been suggested that embryos secretion of a low level of APOA1 is reflecting their viability and metabolic competence (Nyalwidhe et al., 2013). In the present study, APOA1 showed higher abundance in the Cyclic group than the Fragment group, probably due to stimuli of conceptus fragments.

FG subunits, APOA 1, and HPX converged in functional connections with ALB, a protein in the immunological role, transport, and nutrition. ALB plays a fundamental immunological role in the embryo, crossing from maternal blood to uterine fluid, passing into free-living blastocysts aiding its expansion (Crutchfield and Kulangara, 1973). In the present study, ALB showed abundance differences in cyclic and infused mares suggesting post-translational changes by differences in MW and pI, previously reported in follicular fluid (Fahiminiya et al., 2011) and uterine fluid (Bastos et al., 2019) of mares.

ALB has been related to TF in mice's embryo metabolism (McArdle and Priscott, 1984). TF, also known as siderophilin, is a protein that transports iron from storage sites to regions of iron metabolism (MacGillivray et al., 1998). TF was proposed as a protein to protect uterine and conceptus tissues from lipid peroxidation activity, which may occur as a consequence of iron transport via Uteroferrin secretion (Vallet, 1995; Vallet et al., 1996). TF and ALB are negative acute-phase proteins (Gruys et al., 2005) and showed differences in abundance in the Cyclic as in Fragment groups. Probably these proteins were undergone to post-translational changes and acted actively in cyclic and pregnant mare, as reported in many studies (Swegen et al., 2017; Smits et al., 2018; Bastos et al., 2019). Therefore, these proteins are of importance in the maintenance of concepti homeostasis and cellular patterns. Differences in ALB and TF presence with higher abundance in the Cyclic or the Fragment group are probably due to the lack of functional and live conceptus that promotes endocytosis of proteins.

ALB also shows a functional connection with two proteins of defense, SERPINB1, and IGLC7. SERPINB1 protein inhibits the proliferation of leukocytes (Padua and Hansen, 2008; Ulbrich et al., 2009). Serpin's transcripts have been detected in the inner cell mass and trophectoderm of the equine embryo (Iqbal et al., 2014). SERPINB1, associated with the innate immune system, was identified in the endometrial fluid of pregnant mare and the yolk sac (Smits et al., 2018). Uterine SERPINs likely perform diverse biological functions, including direct nutrition of conceptus, growth control, inhibition of proteolytic activities, suppression of local maternal immune system for sustaining pregnancy (Kumar et al., 2013), and control of matrix metalloproteinases-2 expression (Huasong et al., 2015). SERPINB1 belongs to proteins classified as Ovalbumin (Remold-O’Donnell et al., 1992). Ovalbumin has been used in murine as a model to investigate immune mechanisms of allergic lung inflammation, resulting in increased levels of prostaglandins, cytokines, immune cells, and secretions (Moore and Peebles, 2006). Infusion of conceptus fragments reported histomorphometric changes, an increase of glandular secretion, and immune cells in the uterus (Camacho et al., 2018). SERPINB1 signalization may partially mediate these alterations in the early pregnancy (Martínez, 2016; Caballeros et al., 2019; Camozzato et al., 2019).

## Conclusions

Results revealed adhesion, nutrition, endothelial cell proliferation, transport, and immunological tolerance proteins, suggesting that these proteins are functionally important in embryo-maternal communication. Identification of acute-phase proteins and immunoglobulins indicates a great influence of the immunological system. In conclusion, conceptus fragments signalized changes in the protein profile of uterine fluid seven days after ovulation, with the observed at Day 7 in the same cyclic mares.
